# Introducing the Australian Hearing Hub

**DOI:** 10.1177/2331216517722920

**Published:** 2017-07-28

**Authors:** David McAlpine, Catherine McMahon, Harvey Dillon, Greg Leigh, Jim Hungerford, Jim Patrick, Robert Cowan, Louise Dodd

**Affiliations:** 1 Macquarie University, Sydney, Australia; 2 The HEARing Cooperative Research Centre, Melbourne, Australia; 3 National Acoustic Laboratories, Sydney, Australia; 4 Royal Institute of Deaf and Blind Children, Sydney, Australia; 5 The Shepherd Centre, Sydney, Australia; 6 Cochlear Limited, Sydney, Australia; 7 University of Melbourne, Melbourne, Australia

**Keywords:** hearing, communication, Australian Hearing Hub

## Abstract

This special issue contains a collection of papers highlighting the collaborative research taking place at Macquarie University’s Australian Hearing Hub. Included in this introduction is a brief outline of the challenges in the hearing health and communication space and a brief description of the Australian Hearing Hub and its members, alongside an overview of the studies included in this special issue.

## Introduction

It is estimated that 360 million people worldwide have a hearing loss (World Health Organization, 2017), impacting on the health and well-being of individuals, their families, and society at large, if not addressed. The growing global problem of poor hearing health is reflected in Australia. One in six Australians has some form of hearing impairment, and this is projected to rise to one in four by 2050 as our population ages. Despite hearing loss being projected to be in the top 15 leading causes of burden of disease, by 2030, it is often dismissed as simply a normal consequence of the aging process. This failure to engage with the problem of, often treatable, hearing impairment has considerable consequences for how the health and communication needs of our aging societies can be met (Davis et al., 2016). Australia’s highly successful newborn hearing screening and early intervention program is recognized globally, but beyond infancy, as with many developed countries, a life-course perspective of prevention, early detection, and management is lacking. It is widely accepted that new models of hearing health care are required to address the chronic lack of penetration of current therapies and interventions. The field lacks the architecture of other health-care pathways required to implement sustainable change and improvements in clinical practice. Contributing factors to this lack of a clear route to treatment may include the wide range of potential health-care providers for solutions such as hearing aids and the absence of a “standard-of-care” perspective for cochlear implantation in adults. Additionally, advances in basic hearing science and the development of hearing technologies are poorly aligned—recent, rapid progress in understanding biological mechanisms contributing to hearing loss and potential novel therapies for restoring hearing function reside largely outside the current research strategies of most technology companies. Addressing these structural problems in the hearing health-care pathway requires a more coherent focus on key junctures along the development pipeline—from biological investigations of hearing problems, the use of randomize control trials for assessing efficacy, and the development of interventions and therapies in terms of “standard-of-care.”

## Founding of the Australian Hearing Hub

The Australian Hearing Hub is an initiative of Macquarie University and the Commonwealth Government’s Education Investment Fund. It brings together leading researchers and health-care organizations to drive research, education, and innovation to improve the lives of people with hearing, communication, or mental health disorders. Its innovative framework of engagement and collaboration enables researchers, educators, and clinicians to identify and address challenges in hearing health and communication over the life span, conduct joint research projects, and implement effective long-term changes in policy and practice. Major partners in the Australian Hearing Hub include Cochlear Limited, whose world headquarters are located adjacent to the Australian Hearing Hub, Australian Hearing, the largest provider of government-funded hearing services, and its world-recognized research division, the National Acoustic Laboratories. Other key partners include the Royal Institute for Deaf and Blind Children and its associated cochlear-implant program, the Sydney Cochlear Implant Centre, The Shepherd Centre and the Cooperative Research Centre, and The HEARing CRC. Allied with Macquarie’s leadership in the Australian Research Council’s Centre for Cognition and its Disorders, a pan-Australian, indeed global, partnership of research centers dedicated to understanding human cognition and communication, the partners work together to improve communication over the life course.

## Overview of Special Issue

The success of the Australian Hearing Hub collaboration lies not only in the generation of published articles but also advancing clinical best practices and the development of new technologies to improve hearing and communication over the life course. This special issue collates a range of studies from partners within the Australian Hearing Hub. It not only demonstrates the truly collaborative nature of the Hub—each has two or more Hub partners working on the project—but also examines hearing health and communication issues with the specific intent of using evidence-based research to improve the lives of people with hearing loss and communication disorders now and in the future. The Australian Hearing Hub regards evidence-based practice as paramount to developing a successful long-term hearing health-care model. One study (Boisvert et al., 2017) investigates what information audiologists use when discussing rehabilitation choices with clients. This research provides insight into health-care delivery models in clinics. Another examines the psychosocial development of children with hearing loss (Wong et al., 2017). The results suggest that even children who develop good language ability with the help of a hearing aid or cochlear implant may have psychosocial problems if they exhibit difficulties with listening and communicating in everyday environments. Due to increased awareness about the benefits of cochlear implantation and recipients living longer following implantation, the prevalence of reimplantation is increasing. Reis, Boisvert, Looi, and da Cruz (2017) report on speech recognition outcomes before and after cochlear reimplantation to assess the safety and success of this type of surgery. Another study explores the potential benefits of bilateral over unilateral cochlear implants (Rana et al., 2017), demonstrating that both speech comprehension and spatial release from masking are improved in bilaterally implanted adults. The relationship between depression and hearing loss in middle-age compared with older adults is the subject of another study (Keidser & Seeto, 2017) that reveals a potential need to review how best to encourage those individuals in middle age to seek interventions and to support those who do; whether for depression, a hearing problem, or both. Another report investigates how new objective measures for understanding listening effort in background noise might be developed (Miles et al., 2017). The findings of this study will contribute to a growing body of research aimed at developing an objective measure of listening effort.

## Conclusion

The overarching theme of the papers in this special issue is of collaboration—collaboration between universities and government agencies, research partnerships with commercial entities, and engagement with charities and clinical organizations, each with a mission that spans basic science to delivery of hearing health care. This holistic approach to the investigation of hearing and deafness is integral to the increasingly shared vision of the Hub partners as they seek to develop new therapeutic interventions and care pathways. It also sets the agenda for the next stage of the Hub’s development, where joint research frameworks between partners will be employed to leverage significantly more resources for research and innovation than can be achieved separately. Australia’s research-funding ecosystem, its well-resourced health programmes, and the public visibility of iconic Australian “brands” such as Cochlear Limited and Australian Hearing, make this a real possibility. An immediate goal is to ensure hearing becomes Australia’s 10th National Health Priority. From that vantage point, the Australian Hearing Hub can become a model of best practice, pioneering the way in which new therapies and interventions are rapidly assessed for efficacy, and implemented into practice, not just in Australia, but globally.
